# Composition Analysis and Feature Selection of the Oral Microbiota Associated with Periodontal Disease

**DOI:** 10.1155/2018/3130607

**Published:** 2018-11-15

**Authors:** Wen-Pei Chen, Shih-Hao Chang, Chuan-Yi Tang, Ming-Li Liou, Suh-Jen Jane Tsai, Yaw-Ling Lin

**Affiliations:** ^1^Department of Applied Chemistry, Providence University, Taichung City, Taiwan; ^2^Department of Periodontics, Linkou Medical Center, Chang Gung Memorial Hospital, Taoyuan, Taiwan; ^3^Graduate Institute of Dental and Craniofacial Science, Chang Gung University, Taoyuan, Taiwan; ^4^Department of Computer Science and Information Engineering, Providence University, Taichung City, Taiwan; ^5^Department of Medical Laboratory Science and Biotechnology, Yuanpei University of Medical Technology, Hsin-Chu City, Taiwan

## Abstract

Periodontitis is an inflammatory disease involving complex interactions between oral microorganisms and the host immune response. Understanding the structure of the microbiota community associated with periodontitis is essential for improving classifications and diagnoses of various types of periodontal diseases and will facilitate clinical decision-making. In this study, we used a 16S rRNA metagenomics approach to investigate and compare the compositions of the microbiota communities from 76 subgingival plagues samples, including 26 from healthy individuals and 50 from patients with periodontitis. Furthermore, we propose a novel feature selection algorithm for selecting features with more information from many variables with a combination of these features and machine learning methods were used to construct prediction models for predicting the health status of patients with periodontal disease. We identified a total of 12 phyla, 124 genera, and 355 species and observed differences between health- and periodontitis-associated bacterial communities at all phylogenetic levels. We discovered that the genera* Porphyromonas*,* Treponema*,* Tannerella*,* Filifactor*, and* Aggregatibacter *were more abundant in patients with periodontal disease, whereas* Streptococcus*,* Haemophilus*,* Capnocytophaga*,* Gemella*,* Campylobacter*, and* Granulicatella* were found at higher levels in healthy controls. Using our feature selection algorithm, random forests performed better in terms of predictive power than other methods and consumed the least amount of computational time.

## 1. Introduction

The human mouth harbors a complex microbial community, with estimates of up to 700 or more different bacterial species, most of which are commensal and required to maintain the balance of the mouth ecosystem [1]. However, some of the bacteria in the mouth microbiota play important roles in the development of oral diseases, including dental caries and periodontal disease [2]. Periodontal disease and dental caries initiate with the growth of the dental plaque, a biofilm formed by the accumulation of bacteria together with various human salivary glycoproteins and polysaccharides secreted by the microbes [3]. The subgingival plaque, located within the neutral or alkaline subgingival sulcus, is typically inhabited by anaerobic gram-negative bacteria and is responsible for the development of gingivitis and periodontitis. The composition of oral microorganisms depends on multiple factors, including lifestyle (e.g., diet, oral care habits), health (e.g., oral diseases, host immune responses, and genetic susceptibility), and physical location in the oral cavity (tongue or tooth surfaces, as well as supragingival or subgingival sites) [4]. Periodontitis is an inflammatory disease involving a complex interaction between oral microorganisms organized in a biofilm structure and the host immune response. Clinically, periodontitis results in the destruction of tissues that support and protect the tooth and is a major cause of tooth loss in adults [5]. Moreover, periodontitis can also affect systemic health by increasing the risk of atherosclerosis, adverse pregnancy outcomes, rheumatoid arthritis, aspiration pneumonia, and cancer [6–11].

In the past half century, numerous studies have characterized the community composition of the oral microbiota and described the association between periodontitis and pathogenic microorganisms. For example,* Aggregatibacter actinomycetemcomitans*,* Porphyromonas gingivalis*,* Tannerella forsythia*,* Treponema denticola*,* Fusobacterium nucleatum*, and* Prevotella intermedia* have traditionally been considered pathogenic bacteria contributing to periodontitis [5, 12, 13]. Socransky et al. [14] described the role of 5 main microbial complexes in the subgingival biofilm. They reported that red complex species* Porphyromonas gingivalis*,* Treponema denticola*, and* Tannerella forsythia* exhibited a very strong relationship with periodontitis. Subsequently, other association and elimination studies have confirmed the involvement of the three members of the red complex and some members of the orange complex, such as* Prevotella intermedia*,* Parvimonas micra*,* Fusobacterium nucleatum*,* Eubacterium nodatum*, and* Aggregatibacter actinomycetemcomitans*, in the etiology of different periodontal conditions [15]. Additionally, during the past decade, researchers using culture-independent molecular techniques have shown that some representatives of the genera* Megasphaera*,* Parvimonas*,* Desulfobulbus*, and* Filifactor *are more abundant in patients with periodontal diseases, whereas members of* Aggregatibacter*,* Prevotella*,* Selenomonas*,* Streptococcus*,* Actinomyces*, and* Rothia *are more abundant in healthy patients [16–19].

Machine learning is data method that involves finding patterns and making predictions from data based on multivariate statistics, data mining, and pattern recognition. This technology had been used to solved many metagenomic problems, such as operational taxonomic unit (out) clustering [20–24], binning [25–30], taxonomic profiling and assignment [31–35], comparative metagenomics [36–38], and gene prediction [39–42]. In addition to the learning algorithm and the model, the most important component of a learning system is how features are extracted from the domain data, a process known as feature selection. The purposes of feature selection include improving the prediction performance of the predictors, providing faster and more cost-effective predictors, and providing a better understanding of the underlying process that generated the data [43–45]. Feature selection methodology can be categorized into three classes (filter, wrapper, and embedded methods) according to how the feature selection search is combined with the construction of the classification mode. Filter methods estimate the relevance of features by analysis of the intrinsic properties of the data. These methods are computationally simple and fast, can scale to very high-dimensional datasets easily, and are independent of the classification algorithm.

Although much is known about individual species associated with pathogenesis, the global structure of the bacterial community and the microbial signatures of periodontal disease are still poorly understood. In this study, we explored the microbial diversity in the subgingival plaque of healthy patients and patients with periodontal disease using culture-independent molecular methods based on 16S ribosomal DNA cloning. We also compared the bacterial community compositions between healthy patients and patients with periodontal disease and determined the core microbiomes present in these patients. Furthermore, we proposed a novel algorithm for feature selection, and microbes with significant differences were extracted as features and provided to generate feature combinations by applying our algorithm. Using machine learning methods, we built prediction models and found that the health status of patients with periodontal disease could be identified accurately using only a few features.

## 2. Materials and Methods

### 2.1. 16S rRNA Sequence Dataset

In total, 76 samples used for this study were collected from subgingival plaques of 76 unrelated individuals, including 10 patients with severe periodontal disease, 40 patients with moderate periodontal disease, and 26 healthy controls. This study was approved by the Institutional Review Board of Chang Gung Memorial Hospital, Taiwan (approval no. 102-4239B). All patients provided informed consent prior to their enrolment in the study. The oral health statuses of all individuals were determined by a dentist who performed a full-mouth clinical examination that included clinical parameters of periodontal pocket depths, gingival recession, clinical attachment loss, bleeding on probing, tooth mobility, and furcation involvement. These clinical parameters were measured at 6 sites per tooth (mesiobuccal, buccal, distobuccal, distolingual, lingual, and mesiolingual) at all teeth.  Table 1 summarizes the parameters of periodontal pocket depths, bleeding on probing and clinical attachment loss for all of the samples. The classification of periodontitis as slight, moderate, or severe was based on the guidelines of the American Academy of Periodontology [46]. Subjects who had received previous periodontal therapy within two years and recent history of antibiotics taking within last 6 months were excluded.

After sampling, DNA extraction and polymerase chain reaction (PCR) were performed based on methods described by Tang et al. [47]. Following extraction, barcoded PCR amplification was performed with 382-bp amplicons flanking the highly variable V1-V2 region of the 16S rRNA gene sequence [48]. Next-generation sequencing evaluation of oral microbial communities was carried out using an* Illumina MiSeq* Desktop Sequencer after 30 cycles of PCR to enrich the adapter-modified DNA fragments.

### 2.2. Sequence Processing

Paired-end reads sequenced by the Illumina Sequencer were assembled with PEAR software [49]. Using split_libraries.py in QIIME with default parameters [50], assembled reads were demultiplexed, and low-quality reads were filtered. The GoldG database containing the ChimeraSlayer reference database in the Broad Microbiome Utilities [51] was used with UCHIME software [52] for chimera detection and removal. The remaining reads were clustered into OTUs using a de novo OTU selection protocol at the 97% identity level with a USEARCH algorithm [21]. Before clustering sequences, we filtered out all reads that occurred fewer than three times. This reduced the number of unique sequences to a computationally manageable level and potentially reduced the number of errors from sequencing and contamination. The taxonomy associated with each OTU was assigned by blasting a representative sequence of each OTU against the Human Oral Microbiome Database [53] (HOMD). The sequence processing was carried out using our metagenomic analysis platforms [45].

### 2.3. Diversity and Significance Analysis

Sample data stored in the biological observation matrix format were subjected to statistical analysis using R language. We analyzed the sequencing depth of samples prior to downstream analysis using the Shannon index. The main microbes and taxonomic composition of the microbiota in each sample were also estimated. Abundance differences of microbes between sample groups were evaluated using the Kruskal−Wallis test. Four non-phylogeny-based metrics, namely, the observer species, chao 1 metric [54], Ace richness, and Shannon index, were used to evaluate alpha diversity, which represented the amount of diversity contained within communities, by applying the phyloseq R package. UniFrac is a distance metric used for comparing biological communities. Principal coordinate analysis (PCoA) with weighted UniFrac distances was applied to evaluate beta diversity, which represented the amount of diversity shared among communities. Principal component analysis (PCA) was used to characterize the primary microbes contained within communities.

### 2.4. Feature Selection and Machine Learning

In this study, we proposed a method of feature selection for selecting the informative microbes to predict whether an individual suffered from periodontal disease. First, the microbes present at less than 0.5% relative abundance in all samples were ignored, and nonparametric Kruskal*−*Wallis tests were used to detect microorganisms with significantly differential abundance between healthy patients and patients with periodontal disease. Microbes with more significant differential scores were considered features with more information. Then, the prioritized feature combination-generated algorithm shown in Algorithm 1 was adopted to produce the feature combinations composed by these more informative features.

In prioritized order, the feature combinations were applied to build classifiers with machine learning algorithms, such as deep learning, support vector machine (SVM), random forests, and logistic regression. We picked 80% of samples from both healthy and disease cases to train the prediction model, and the remaining cases were used for testing. The prediction ability of each feature combination was evaluated by calculating the average accuracy from 10 predictions with different training and testing sample sets. Here, we selected 10 of the most significant features having* p* values between 3.27E-11 and 7.77E-9. In total, 1,023 feature combinations were evaluated for their prediction ability using deep learning, SVM, random forest, and logistic regression methods. These machine learning algorithms were supported by the R packages H_2_O, e1071, randomForest, and stats, respectively. We considered the radial basis function kernel for SVM. Parameters for each machine learning algorithm were tuned using grid search, and the parameters that obtained better accuracy were adopted for training prediction models.

## 3. Results and Discussion

### 3.1. Sample Sequencing and Identification

In total, 76 subgingival plaque samples from 76 unrelated individuals were divided into three classes according to their periodontal health status, i.e., healthy (H), severe periodontitis (SP), and moderate periodontitis (MP). Following DNA extraction and barcoded PCR amplification, these samples were sequenced, generating a total of 7,530,767 sequences. After filtering and trimming, 6,170,984 sequences remained, and there were 481 OTUs in all samples (481 and 429 in diseased and healthy samples, respectively). Due to variations in the number of sequences among samples, the total sequence reads within a sample was normalized to the relative abundance for subsequent analyses.

### 3.2. Taxonomic Composition of the Human Oral Microbiota

Table 2 summarizes the dominant microbes in the human oral microbial communities. In the experimental results, the microbial communities included 12 different phyla:* Bacteroidetes*,* Firmicutes*,* Fusobacteria*,* Proteobacteria*,* Spirochaetes*,* Actinobacteria*,* Candidate division TM7*,* Synergistetes*,* Fusobacteria*,* Candidate division SR1*,* Gracilibacteria*, and* Chloroflexi*.* Bacteroidetes *(37%) was the most abundant phylum in the human oral microbiota. The major genera consisted of previously characterized oral bacteria, including* Prevotella *(13.56%),* Fusobacterium *(11.30%),* Porphyromonas* (10.94%),* Treponema* (8.86%),* Streptococcus *(6.52%),* Leptotrichia *(4.76%), and* Capnocytophaga* (3.64%). In summary, there were 25 classes, 40 orders, 66 families, 124 genera, and 355 species at each taxonomic level.

**Figure 1:**
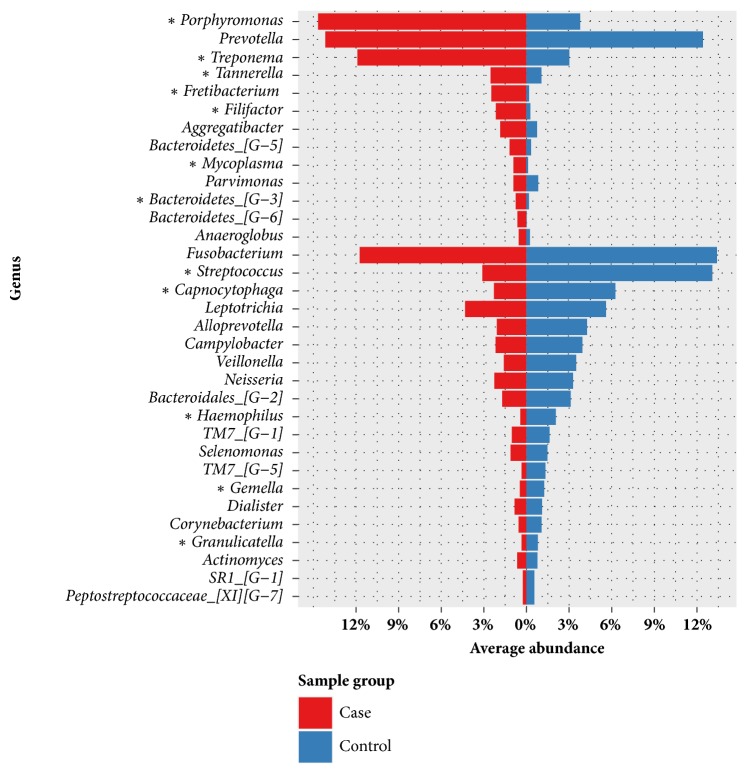
Microbial compositions of samples from healthy patients and patients with periodontitis at the genus level. The abundances were calculated by averaging the relative abundances in samples from healthy patients and patients with periodontitis. Only genera with > 0.5% abundance in at least one sample were included. Genera with significant differences in abundance between sample groups are indicated with asterisks (*∗*) (*p* value < 0.0001).

**Figure 2:**
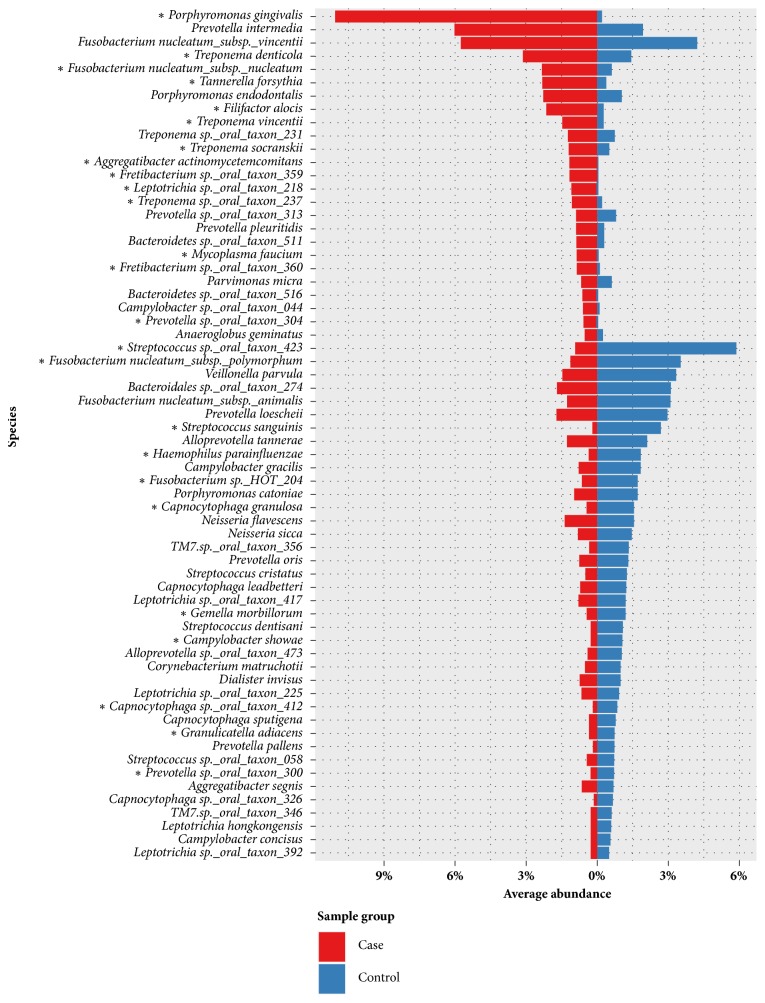
Microbial compositions of samples from healthy patients and patients with periodontitis at the species level. The abundances were calculated by averaging the relative abundances in samples from healthy patients and patients with periodontitis. Only species with* >* 0.5% abundance in at least one sample are shown. Species with significant differences in abundance between sample groups are indicated with asterisks (*∗*) (*p* value < 0.0001).

In comparison of the compositions of microbial communities between healthy patients and patients with periodontitis, we found that the spectra of microbial communities differed. In healthy samples, the dominant genera were* Streptococcus *(13.09%),* Prevotella *(12.43%),* Fusobacterium *(11.70%),* Capnocytophaga *(6.25%),* Leptotrichia *(5.60%),* Alloprevotella *(4.26%),* Campylobacter *(3.94%),* Porphyromonas *(3.78%),* Veillonella *(3.49%), and* Neisseria *(3.27%); however, in patients with periodontal disease, the dominant genera were* Porphyromonas *(14.67%),* Prevotella *(14.16%),* Treponema *(11.90%),* Fusobacterium *(11.09%),* Leptotrichia *(4.32%), and* Streptococcus *(3.10%). At the species level,* Streptococcus *sp.* oral taxon 423 *(0.2-36%) was the most abundant species in healthy patients, whereas* Porphyromonas gingivalis *(0-31%) was the most abundant species in patients with periodontitis.  Table 3 compares the dominant microbes between healthy patients and patients with periodontitis at each taxonomic level. The genus and species level taxonomic compositions between healthy patients and patients with periodontitis are shown in Figures 1 and 2.* Streptococcus *was more abundant in samples from all healthy individuals but decreased in samples from patients with periodontitis. Additionally,* Porphyromonas* and* Treponema *were more abundant in patients with periodontitis but decreased significantly in samples from healthy individuals. In total, 25 species were identified with significantly different abundances between sample groups;* Porphyromonas gingivalis* was the species with the most significantly differential abundance between samples from healthy patients and patients with periodontitis (*p* value = 2.41E-9).

Overall, our findings were largely comparable to those of previous studies [14, 55–61], indicating that species such as* Porphyromonas gingivalis*,* Treponema denticola*,* Tannerella forsythia*,* Filifactor alocis*,* Treponema socranskii*,* Aggregatibacter actinomycetemcomitans*,* Treponema vincentii*, and* Mycoplasma faucium *were significantly enriched in samples from patients with periodontitis. Furthermore, we found a set of species, including* Streptococcus sanguinis*,* Haemophilus parainfluenzae*,* Capnocytophaga granulosa*,* Gemella morbillorum*,* Campylobacter showae*, and* Granulicatella adiacens*, were significantly enriched in samples from healthy individuals.

**Figure 3:**
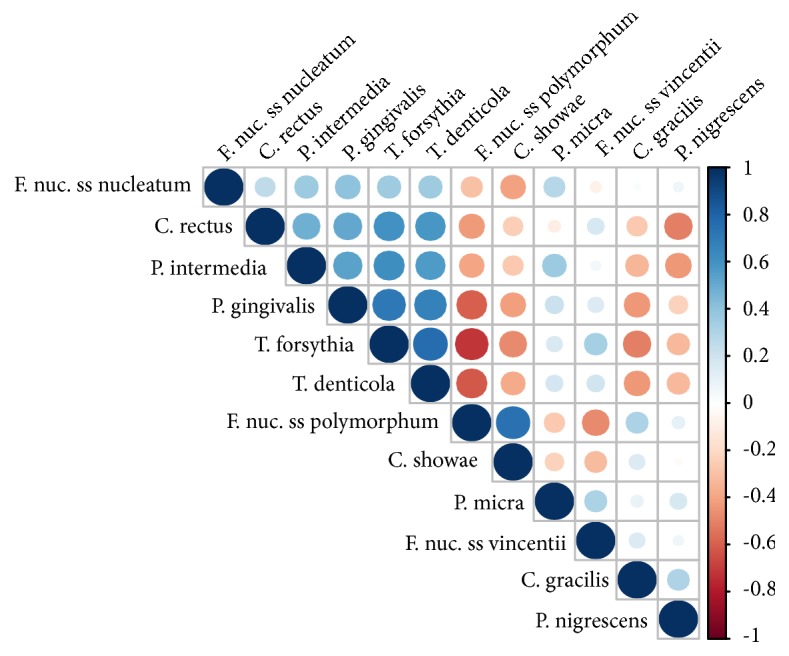
The relationships among species were evaluated using Spearman's rank correlation coefficient.

Several studies have described the bacterial communities in patients with periodontitis and healthy control participants using metagenomics [16–19, 61–63]. The dominant microorganisms associated with periodontitis and the healthy state were largely consistent in those studies; however, we observed several discrepancies. First, in addition to common diseased-associated microorganisms, such as* Porphyromonas gingivalis*,* Treponema denticola*,* Tannerella forsythia*,* Filifactor alocis*, and* Aggregatibacter actinomycetemcomitans*, we also found that the species* Mycoplasma faucium *was significantly enriched in samples from patients with periodontal disease. There were 26 samples that contained this species at greater than 0.5% abundance, and only one of these samples was derived from a healthy patient. The average relative abundance of* Mycoplasma faucium *was 0.59% in all samples (0.04% and 0.87% in samples from healthy patients and patients with periodontal disease, respectively) and was up to 4.85% in one diseased sample. Although this is a rare bacterium in the normal microbiota of the human oropharynx, some reports have identified this pathogen in brain abscesses [64, 65]. Additionally, Liu et al. [61] characterized the genomes of key players in the subgingival microbiota in patients with periodontitis, including an unculturable* TM7 *organism. They also demonstrated that* TM7 *organisms were significantly enriched in samples from patients with periodontitis. In our study, 49 of 76 samples contained* TM7 *bacteria at greater than 1% abundance (average abundance of 2.1% in all samples). In samples from healthy patients and patients with periodontitis, the average abundances were 3.2% and 1.49%, respectively. However, significant enrichment was not observed in samples from patients with periodontitis. Furthermore, we found that the subspecies* Fusobacterium nucleatum *subsp.* polymorphum*, which is related to periodontal disease and is the member of the orange cluster described by Socransky et al. [14], is more abundant in healthy patients. In our results, the average abundances were 3.52% and 1.13% in samples from healthy patients and patients with periodontitis, respectively. This situation also can be observed in other three species, including* Campylobacter gracilis*,* Campylobacter rectus, *and* Campylobacter showae*. This discrepancy could be explained by geographic variability [66] or by differences in the depths of the pockets sampled [14], as well as the sample size and the DNA analytic bias [67]. Finally, Spearman's rank correlation coefficient was computed to assess association between each pair of species associated with periodontal disease.  Figure 3 shows that a very strong relationship exhibited among species* Porphyromonas gingivalis*,* Treponema denticola*, and* Tannerella forsythia*.

**Figure 4:**
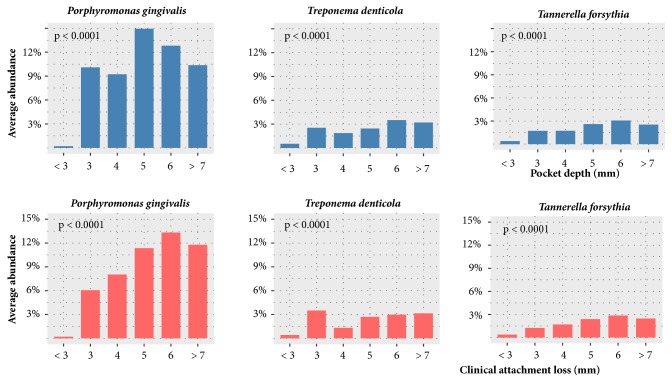
Relationships of the average abundance of three species to selected pocket depths and clinical attachment loss levels. Significance of differences among pocket depth levels was tested using the Kruskal-Wallis test.

In our study, there are 25 bacterial species with significantly different abundances between healthy patients and patients with periodontitis. The relationships of these species to pocket depth and clinical attachment loss were examined.  Figure 4 shows that three species,* Porphyromonas gingivalis*,* Treponema denticola*, and* Tannerella forsythia, *exhibited a very strong relationship with pocket depth and clinical attachment loss. For instance, the three species increased in abundance with increasing pocket depth and clinical attachment loss. The abundances of those species among different level of pocket depth and clinical attachment loss were different significantly. However, it should be noted that not only oral microorganisms but also others factors, such as supragingival plaque, would affect the pocket depth and clinical attachment loss [68].

### 3.3. Diversity of Bacterial Community Profiles

**Figure 5:**
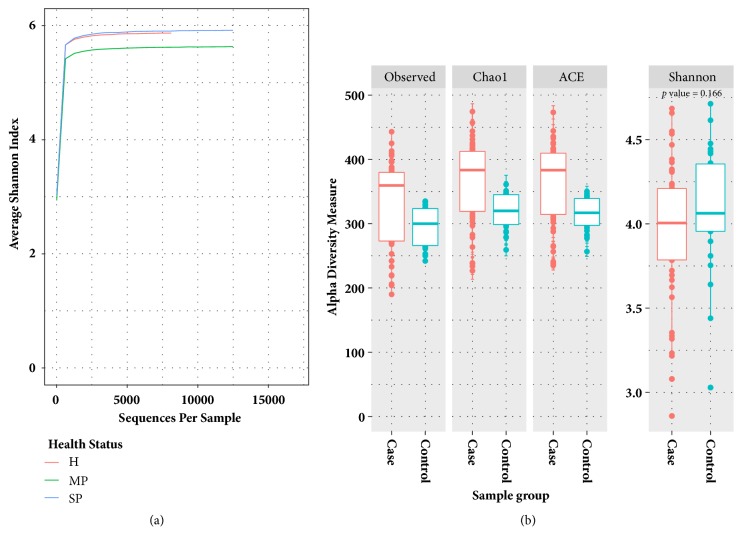
(a) The sequencing depths measured by average scores from the Shannon index reached a plateau when the sequence number exceeded 5,000. (b) Alpha-diversity metrics (richness and Shannon index) were employed to measure the microbial communities of samples from healthy patients and patients with periodontitis. The average richness of microbes was higher in patients with periodontal disease than in healthy patients; however, the microbial communities of healthy patients exhibited higher Shannon indexes.

To evaluate the alpha diversity of the microbial communities, Shannon index curves scores and richness metrics (Observed, Chao1, and ACE) were applied, as shown in Figure 5. As depicted in Figure 5(a), the Shannon diversity index curves clearly reached plateau levels after the sequence number exceeded 5,000 in all three health statuses, indicating that the microbial composition for each health status was well represented by the sequencing depth. As shown in Figure 5(b), the average richness measured by Observed, Chao1, and Ace indexes was higher in samples from patients with periodontitis than in samples from healthy individuals; however, these results were in contrast to the results from the Shannon diversity index. Thus, the relative abundance of each microbe was more balanced in samples from healthy individuals than in samples from patients with periodontal disease, and there were more microbes with low relative abundance in samples from patients with periodontitis.

**Figure 6:**
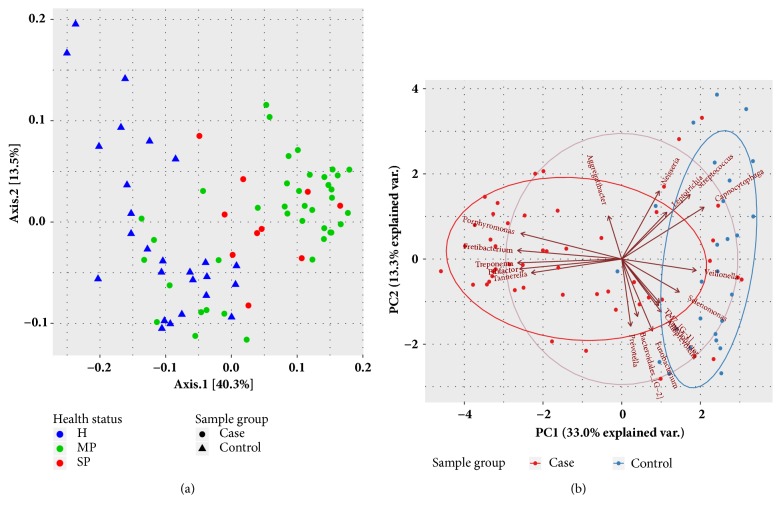
(a) Principal coordinate analysis (PCoA) with weighted UniFrac distance matrixes for bacterial communities associated with the three health statuses. (b) Principal component analysis (PCA) of the dominant genera between samples from healthy patients and patients with periodontitis. Only genera with ≥ 1% mean relative abundance across all samples are shown.

To further explore the relationships between bacterial communities in healthy patients and patients with periodontal disease, PCoA was performed (Figure 6(a)). Analysis of beta diversity based on the weighted UniFrac distances showed greater concentration in diseased samples than in healthy samples. In other words, the microbial compositions of diseased samples were more similar to each other. As shown in Figure 6(b), PCA of microbial communities revealed that the core genera in healthy samples included* Streptococcus*,* Capnocytophaga*,* Campylobacter*,* Veillonella*,* Alloprevotella*,* TM7_[G-1]*,* Leptotrichia*, and* Selenomonas*, whereas those in samples from patients with periodontitis were* Filifactor*,* Treponema*,* Fretibacterium*,* Porphyromonas*, and* Tannerella*.

### 3.4. Machine Learning and Feature Selection

**Figure 7:**
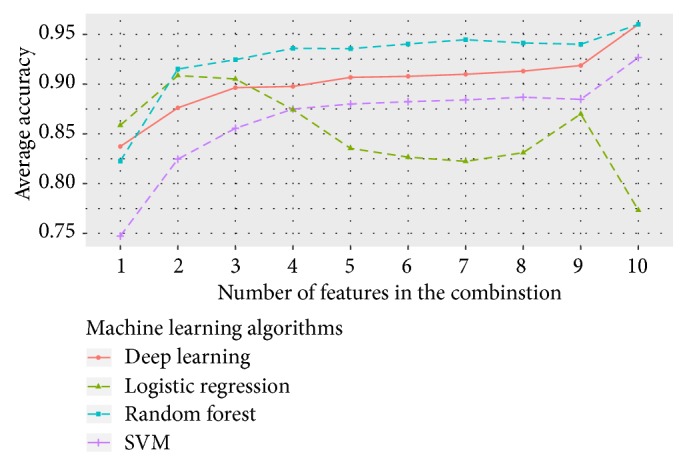
Average accuracies of different numbers of features.

Before applying the machine learning algorithm to classify samples, it is necessary to select the features from the samples and train prediction models.  Table 4 lists features with difference scores* p* < 1.E-07. Based on significant differences between healthy patients and patients with periodontitis, we selected the top 10 microbes with more information as features. In total, 1,023 combinations of selected features were generated by our algorithm. All feature combinations were evaluated by SVM, random forest, logical regression, and deep learning machine learning methods, and the average accuracies were 0.88, 0.93, 0.85, and 0.90, respectively.  Figure 7 shows the performance of each machine learning method. In general, the accuracy of prediction increased slightly with the number of features used, except in logistic regression. From our results, we found that random forests had better predictive ability than the other methods. Applying combinations consisting of* Peptoniphilaceae *sp*. oral taxon 113*,* Streptococcus sanguinis*,* Mollicutes *sp.* oral taxon 906*,* Aggregatibacter actinomycetemcomitans*,* Porphyromonas gingivalis*,* Peptostreptococcaceae *sp.* oral taxon 950*, and* Lachnospiraceae *sp.* oral taxon 500 *or* Stomatobaculum *sp.* oral taxon 373*,* Desulfobulbus *sp.* oral taxon 041*,* Peptoniphilaceae *sp.* oral taxon 113*,* Streptococcus sanguinis*,* Aggregatibacter actinomycetemcomitans*,* Porphyromonas gingivalis*, and* Leptotrichia *sp.* oral taxon 218 *showed that random forests could predict the health status of samples accurately. The feature combinations having average accuracies of more than 0.94 are reported in Table 5.

According to previous studies, Caruana et al. [69, 70] proposed that the random forest method showed better accuracy in high-dimensional and large-scale data than neural nets, SVM, and logistic regression. In this study, we found that the random forest method was more suitable for small-scale data than other methods. In contrast, deep learning approaches led to good performance, but required long computation times and large amounts of memory, particularly when the hidden layer size was increased.

## 4. Conclusions

With the development of high-throughput DNA sequencing technology, the limitations associated with difficult culture of many microbes that populate the oral cavity can be overcome, facilitating the analysis of bacterial community composition. Using 16S rRNA sequencing of subgingival samples from 50 individuals with periodontitis and 26 periodontally healthy controls, we determined the diversity of and differences in community compositions. Moreover, we identified microbes associated with good health and periodontal disease and provided a machine learning method for finding patterns and making predictions for oral microbiota associated with periodontal disease.

Our results showed that there was a higher diversity of microbes in samples from patients with periodontal disease than in samples from healthy patients. Importantly, the core microbes in healthy patients were different significantly from those in patients with periodontitis. We also found that bacterial communities associated with healthy and diseased states were highly different in PCA and PCoA, and the compositions of microorganisms were more similar to each other in samples from patients with periodontal disease than in samples from healthy individuals.

We proposed a novel feature selection method and investigated the potential of machine learning approaches for determination of health status based on oral metagenomics data. By using nonparametric Kruskal*−*Wallis tests to assess the significance of each microorganism, we selected significant microbes to generate prioritized feature combinations by our algorithm. The performances of four machine learning approaches were evaluated with these feature combinations, and random forests showed the best performance (average accuracy of 0.93 from 1,023 feature combinations), followed by deep learning, SVM, and logistic regression. Using machine learning methods, training models could accurately predict the health status of samples by examining fewer features. According to our observations, the accuracy of prediction generally increased slightly with the number of features used, except for logistic regression. Notably, certain combinations composed of fewer features showed better accuracy than combinations composed of all selected features. These combinations of features may only apply to our dataset. However, the results implied that a few related features may have better predictive ability than multiple independent features. Therefore, in order to improve the prediction accuracy of the model, it is essential to identify the most informative features. Due to limitations in funding, time, and ethical considerations, it is not easy to obtain large numbers of oral samples from patients with periodontitis. Although insufficient and incomplete samples could easily lead to bias and variance in training models, our study still provided an important basis for further studies.

Periodontitis is a chronic inflammatory disease involving complex interactions between the oral microorganisms and the host immune response. In addition to the individual species associated with pathogenesis, the system-level mechanisms underlying the transition from a healthy state to a diseased state are key points for studying periodontal disease. Thus, in our future studies, we aim to elucidate the global genetic, metabolic, and ecological changes associated with periodontitis and identify the pathogenic features of constructing machine learning models. Rapid molecular techniques and machine learning methods capable of identifying periodontal bacteria with great accuracy may eventually provide improved classification and diagnosis of various types of periodontal diseases and aid significantly in clinical decision-making.

## Figures and Tables

**Algorithm 1:**
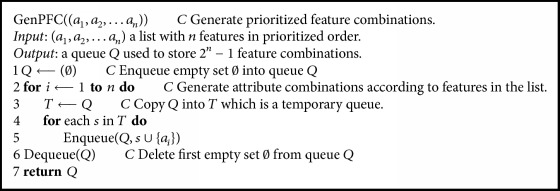
The prioritized feature combination-generated algorithm was used to generate all combinations of selected features in prioritized order. As an example, when *n* equals four, the generated list will be (1000, 0100, 1100, 0010, 1010, 0110, 1110, 0001, 1001, 0101, 1101, 0011, 1011, 0111, 1111). Each element is a combination and denotes whether the four features were selected in that combination (e.g., the combination containing the first and third features is represented as 1010).

**Table 1:** Clinical characteristics of studied subjects. Clinical attachment loss and probing depth were measured in mm and represent the mean for all collected sites in the oral cavity of studied subjects.

Characteristics	Healthy	Moderate periodontitis	Severe periodontitis
Probing depth (mean ± s.d.)	1.3 ± 0.6	5.0 ± 1.3	7.9 ± 0.7
Clinical attachment loss (mean ± s.d.)	1.6 ± 0.7	5.7 ± 1.5	8.6 ± 1.1
% sites with bleeding on probing (mean ± s.d.)	2.8 ± 1.8	68.3 ± 23.2	79.7 ± 17.5

**Table 2:** Dominant microbes of the human oral microbiota at each taxonomic level.

Phylum	Class	Order	
*Bacteroidetes*	37.41% *Bacteroidia*	31.71% *Bacteroidales*	31.71%
*Firmicutes*	20.82% *Fusobacteria*	16.06% *Fusobacteriales*	16.06%
*Fusobacteria*	16.06% *Spirochaetia*	8.86% *Spirochaetales*	8.86%
*Proteobacteria*	9.30% *Bacilli*	7.83% *Lactobacillales*	7.06%
*Spirochaetes*	8.86% *Clostridia*	6.78% *Clostridiales*	6.78%
*Actinobacteria*	2.38% *Negativicutes*	5.21% *Selenomonadales*	5.21%

Family	Genus	Species	

*Prevotellaceae*	16.39% *Prevotella*	13.56% *Porphyromonas gingivalis*	7.30%
*Porphyromonadaceae*	12.96% *Fusobacterium*	11.30% *Fusobacterium nucleatum_subsp._vincentii *	5.23%
*Fusobacteriaceae*	11.30% *Porphyromonas*	10.94% *Prevotella intermedia*	4.62%
*Spirochaetaceae*	8.86% *Treponema*	8.86% *Streptococcus sp._oral_taxon_423*	2.62%
*Streptococcaceae*	6.52% *Streptococcus*	6.52% *Bacteroidales sp._oral_taxon_274*	2.18%
*Veillonellaceae*	5.21% *Leptotrichia*	4.76% *Prevotella loescheii*	2.15%

**Table 3:** Dominant microbes of the oral microbiota between healthy patients and patients with periodontitis at each taxonomic level.

	Healthy patients	Patients with periodontitis	
**Phylum**			
	*Bacteroidetes*	31.93% *Bacteroidetes*	40.26%
	*Firmicutes*	26.90% *Firmicutes*	17.66%
	*Fusobacteria*	17.31% *Fusobacteria*	15.42%
	*Proteobacteria*	11.81% *Spirochaetes*	11.90%
	*Actinobacteria*	3.36% *Proteobacteria*	7.99%
	*Saccharibacteria*	3.20% *Synergistetes*	2.50%
**Class**			
	*Bacteroidia*	24.76% *Bacteroidia*	35.32%
	*Fusobacteria*	17.31% *Fusobacteria*	15.42%
	*Bacilli*	15.23% *Spirochaetia*	11.90%
	*Negativicutes*	6.71% *Clostridia*	7.93%
	*Flavobacteriia*	6.67% *Negativicutes*	4.43%
	*Clostridia*	4.57% *Bacilli*	3.98%
**Order**			
	*Bacteroidales*	24.76% *Bacteroidales*	35.32%
	*Fusobacteriales*	17.31% *Fusobacteriales*	15.42%
	*Lactobacillales*	13.94% *Spirochaetales*	11.90%
	*Selenomonadales*	6.71% *Clostridiales*	7.93%
	*Flavobacteriales*	6.67% *Selenomonadales*	4.43%
	*Clostridiales*	4.57% *Lactobacillales*	3.48%
**Family**			
	*Prevotellaceae*	16.69% *Porphyromonadaceae*	17.19%
	*Streptococcaceae*	13.09% *Prevotellaceae*	16.24%
	*Fusobacteriaceae*	11.70% *Spirochaetaceae*	11.90%
	*Veillonellaceae*	6.71% *Fusobacteriaceae*	11.09%
	*Flavobacteriaceae*	6.67% *Veillonellaceae*	4.43%
	*Leptotrichiaceae*	5.61% *Leptotrichiaceae*	4.32%
**Genus**			
	*Streptococcus*	13.09% *Porphyromonas*	14.67%
	*Prevotella*	12.43% *Prevotella*	14.16%
	*Fusobacterium*	11.70% *Treponema*	11.90%
	*Capnocytophaga*	6.25% *Fusobacterium*	11.09%
	*Leptotrichia*	5.60% *Leptotrichia*	4.32%
	*Alloprevotella*	4.26% *Streptococcus*	3.10%
**Species**			
	*Streptococcus sp._oral_taxon_423*	5.88% *Porphyromonas gingivalis*	11.01%
	*Fusobacterium nucleatum_subsp._vincentii*	4.22% *Prevotella intermedia*	6.02%
	*Fusobacterium nucleatum_subsp._polymorphum*	3.52% *Fusobacterium nucleatum_subsp.*_*vincentii*	5.76%
	*Veillonella parvula*	3.33% *Treponema denticola*	2.68%
	*Bacteroidales sp.*_*oral*_*taxon*_*274*	3.11% *Fusobacterium nucleatum_subsp.*_*nucleatum*	2.34%
	*Fusobacterium nucleatum_subsp._animalis*	3.09% *Tannerella forsythia*	2.32%

**Table 4:** Features with significant differences between healthy patients and patients with periodontitis. Correlation coefficients and *p *values were determined by Spearman's rank correlation coefficient and Kruskal*−*Wallis tests, respectively. Negative correlations indicated that the features were observed more often in patients with periodontitis than in healthy patients.

No	Feature (Species)	Correlation coefficient	*p*
1	*Stomatobaculum sp._oral_taxon_373*	-0.766029754	3.27E-11
2	*Desulfobulbus sp._oral_taxon_041*	-0.74877058	8.90E-11
3	*Peptoniphilaceae sp._oral_taxon_113*	-0.723418056	3.73E-10
4	*Streptococcus sanguinis*	0.71684624	5.36E-10
5	*Mollicutes sp._oral_taxon_906*	-0.709369416	8.08E-10
6	*Aggregatibacter actinomycetemcomitans*	-0.686608198	2.74E-09
7	*Porphyromonas gingivalis*	-0.683993685	3.15E-09
8	*Peptostreptococcaceae sp._oral_taxon_950*	-0.681489164	3.59E-09
9	*Lachnospiraceae sp._oral_taxon_500*	-0.670324546	6.43E-09
10	*Leptotrichia sp._oral_taxon_218*	-0.666642231	7.77E-09
11	*Bosea vestrisii*	0.665468802	8.26E-09
12	*Filifactor alocis*	-0.656797473	1.29E-08
13	*Mycoplasma faucium*	-0.641322841	2.79E-08
14	*Prevotella sp._oral_taxon_304*	-0.638587976	3.20E-08
15	*Fretibacterium sp._oral_taxon_359*	-0.632290825	4.36E-08
16	*Bergeyella sp._oral_taxon_322*	0.630961524	4.65E-08
17	*Tannerella forsythia*	-0.628346704	5.28E-08
18	*Peptostreptococcus indolicus*	-0.626504998	5.77E-08
19	*Johnsonella sp._oral_taxon_166*	-0.622396393	7.04E-08
20	*Peptostreptococcaceae [Eubacterium]_saphenum*	-0.616735679	9.24E-08

**Table 5:** Feature combinations and their predictive accuracies with different machine learning methods. Only feature combinations with more than 0.94 average accuracy are shown. DL, RF, and LR represent deep learning, random forests, and logistic regression, respectively.

**Feature combination**	**DL**	**RF**	**SVM**	**LR**	**Average accuracy**
*Stomatobaculum sp._oral_taxon_373*	0.967	0.973	0.960	0.933	0.958
*Peptoniphilaceae sp._oral_taxon_113*					

*Desulfobulbaceae sp._oral_taxon_041*	0.933	0.960	0.973	0.947	0.953
*Peptoniphilaceae sp._oral_taxon_113 *					
*Aggregatibacter actinomycetemcomitans*					
*Lachnospiraceae sp._oral_taxon_500 *					
*Leptotrichia sp._oral_taxon_218 *					

*Stomatobaculum sp._oral_taxon_373 *	0.933	0.973	0.960	0.947	0.953
*Streptococcus sanguinis*					
*Aggregatibacter actinomycetemcomitans*					

*Desulfobulbaceae sp._oral_taxon_041 *	0.973	0.967	0.933	0.927	0.950
*Mollicutes sp._oral_taxon_906 *					
*Porphyromonas gingivalis *					
*Aggregatibacter actinomycetemcomitans*					
*Peptostreptococcaceae sp._oral_taxon_950*					

*Stomatobaculum sp._oral_taxon_373 *	0.947	0.953	0.907	0.987	0.948
*Streptococcus sanguinis*					
*Mollicutes sp._oral_taxon_906*					
*Porphyromonas gingivalis*					
*Aggregatibacter actinomycetemcomitans*					

*Stomatobaculum sp._oral_taxon_373 *	0.960	0.967	0.947	0.913	0.947
*Peptoniphilaceae sp._oral_taxon_113 *					
*Aggregatibacter actinomycetemcomitans*					
*Leptotrichia sp._oral_taxon_218 *					

*Desulfobulbaceae sp._oral_taxon_041 *	0.933	0.973	0.933	0.947	0.947
*Peptoniphilaceae sp._oral_taxon_113 *					
*Aggregatibacter actinomycetemcomitans*					
*Leptotrichia sp._oral_taxon_218 *					

*Stomatobaculum sp._oral_taxon_373 *	0.967	0.933	0.953	0.933	0.947
*Peptoniphilaceae sp._oral_taxon_113 *					
*Mollicutes sp._oral_taxon_906 *					

*Peptoniphilaceae sp._oral_taxon_113 *	0.960	0.987	0.867	0.967	0.945
*Streptococcus sanguinis*					
*Aggregatibacter actinomycetemcomitans*					

*Stomatobaculum sp._oral_taxon_373 *	0.920	0.947	0.967	0.947	0.945
*Aggregatibacter actinomycetemcomitans*					
*Peptostreptococcaceae sp._oral_taxon_950*					

*Stomatobaculum sp._oral_taxon_373 *	0.967	0.967	0.953	0.893	0.945
*Peptoniphilaceae sp._oral_taxon_113 *					
*Porphyromonas gingivalis *					
*Aggregatibacter actinomycetemcomitans*					

## Data Availability

The raw sequences of human oral subgingival plaque samples were deposited at the NCBI Sequence Read Archive under the Bioproject Accession no. PRJNA437129.
